# Intensive Care Syndrome: Promoting Independence and Return to Employment (InS:PIRE). Early evaluation of a complex intervention

**DOI:** 10.1371/journal.pone.0188028

**Published:** 2017-11-29

**Authors:** Joanne McPeake, Martin Shaw, Theodore J. Iwashyna, Malcolm Daniel, Helen Devine, Lyndsey Jarvie, John Kinsella, Pamela MacTavish, Tara Quasim

**Affiliations:** 1 University of Glasgow, School of Medicine, Dentistry and Nursing, Glasgow, Scotland, United Kingdom; 2 NHS Greater Glasgow and Clyde, Glasgow Royal Infirmary, Intensive Care Unit, Glasgow, United Kingdom; 3 NHS Greater Glasgow and Clyde, Glasgow Royal Infirmary, Clinical Physics Department, Glasgow, Scotland, United Kingdom; 4 Center for Clinical Management Research, VA Ann Arbor Health System, Ann Arbor, Michigan, United States of America; 5 Department of Internal Medicine, Division of Pulmonary and Critical Care, University of Michigan, Ann Arbor, Michigan, United States of America; Azienda Ospedaliero Universitaria Careggi, ITALY

## Abstract

**Background:**

Many patients suffer significant physical, social and psychological problems in the months and years following critical care discharge. At present, there is minimal evidence of any effective interventions to support this patient group following hospital discharge. The aim of this project was to understand the impact of a complex intervention for ICU survivors.

**Methods:**

Quality improvement project conducted between September 2014 and June 2016, enrolling 49 selected patients from one ICU in Scotland. To evaluate the impact of this programme outcomes were compared to an existing cohort of patients from the same ICU from 2008–2009. Patients attended a five week peer supported rehabilitation programme. This multidisciplinary programme included pharmacy, physiotherapy, nursing, medical, and psychology input. The primary outcome in this evaluation was the EQ-5D, a validated measure of health-related quality of life. The minimally clinically important difference (MCID) in the EQ-5D is 0.08. We also measured change in self-efficacy over the programme duration. Based on previous research, this study utilised a 2.4 (6%) point change in self-efficacy scores as a MCID.

**Results:**

40 patients (82%) completed follow-up surveys at 12 months. After regression adjustment for those factors known to impact recovery from critical care, there was a 0.07–0.16 point improvement in quality of life for those patients who took part in the intervention compared to historical controls from the same institution, depending on specific regression strategy used. Self-efficacy scores increased by 2.5 points (6.25%) over the duration of the five week programme (p = 0.003), and was sustained at one year post intervention. In the year following ICU, 15 InS:PIRE patients returned to employment or volunteering roles (88%) compared with 11 (46%) in the historical control group (p = 0.15).

**Conclusions and relevance:**

This historical control study suggests that a complex intervention may improve quality of life and self-efficacy in survivors of ICU. A larger, multi-centre study is needed to investigate this intervention further.

## Background

Patients who survive a critical care admission often suffer persistently low quality of life and high ongoing medical costs [1–4]. ‘Post Intensive Care Syndrome’ includes physical morbidity such as chronic pain and poor mobility, anxiety, Post-Traumatic Stress Disorder, cognitive problems and an abundance of social sequelae [5–13]. Early mobilisation and timely recognition of delirium may improve outcomes for this patient cohort during the Intensive Care Unit (ICU) stay [14–15]. However, recent investigations have demonstrated that existing post-ICU in-patient and outpatient interventions have had minimal impact on functional outcomes [16–18].

Peer support as a strategy for recovery has been shown to be beneficial in several disease processes [19–21]. Evidence from qualitative work within critical care has also demonstrated that peer support may have a beneficial impact, with the potential of reducing social isolation for both patients and caregivers [2,22]. This has not previously been tested within the critical care rehabilitation environment in the context of an interventional study.

Intensive Care Syndrome: Promoting Independence and Return to Employment (InS:PIRE) is a five week, peer support programme co-produced with patients and caregivers. Within the InS:PIRE programme, patients receive individual and groups sessions with nurses, medical staff, physiotherapists, psychologists, pharmacists and community organisations. The main aim of InS:PIRE is to empower patients to take control of their health and wellbeing. No such intervention has been rigorously evaluated prior to this. We therefore sought to conduct an initial evaluation of the InS:PIRE program with regard to three endpoints: change in EQ-5D measured by a health utility score over time (and compared to a group of patients discharged from the same ICU between 2008–2009); change in reported self-efficacy over time; and return-to-work.

## Methods

InS:PIRE took place in a 20-bed mixed medical/surgical critical care unit in Glasgow Royal Infirmary (GRI). GRI is a tertiary referral centre for burn and pancreatic care and is situated in an area of high socioeconomic deprivation, with 42% of the most deprived geographical areas in Scotland residing in the GRI catchment area [23].

InS:PIRE was undertaken as part of a quality improvement initiative within the ICU. Ethics approval was sought and waived by our hospital research and development department. The Caldicott Guardian within NHS Greater Glasgow and Clyde also reviewed the proposal. Ethics Approval was obtained for the historical control data, which was collected between 2008 and 2009 (West of Scotland Research Ethics Committee 3, 10/S0701/62).

Patients attended InS:PIRE between September 2014 and June 2015. Six month and one year follow up occurred between February 2015 and June 2016. Patients were eligible to attend if they were of working age (under 65 years) and had either a level-three stay of greater than 72 hours, or a level-two stay of greater than two weeks. The term “level-three” refers to the UK Intensive Care Society definition of ICU patients. Level-three patients require multiple organ support or invasive respiratory support only [24]. Level-two patients are those patients requiring more detailed observation or interventions, including support from a single failing organ system, or post-operative care and those stepping down from higher levels of care. Exclusion criteria for this evaluation were limited to those patients with significant brain injuries and those patients under 18.

Patients were invited to attend InS:PIRE between 6–20 weeks post hospital discharge. Several patients who were further along the recovery trajectory requested to attend the intervention. Caregivers were also encouraged to attend with patients. There was no specific inclusion criteria for caregivers invited to InS:PIRE. Patients could still attend without a caregiver.

### Intervention

Intensive Care Syndrome: Promoting Independence and Return to Employment (InS:PIRE) is a five week, multi-disciplinary, peer supported rehabilitation programme for ICU survivors and caregivers. InS:PIRE is a complex intervention which was co-produced with staff, patients and caregivers. InS:PIRE took place in the hospital setting.

Over the five week programme, patients and caregivers undertook a weekly physiotherapy class. The aim of this class was to improve physical functioning. It also created a forum for patient peer support and offered the opportunity for patients and caregivers to share experiences about recovery. During the first three weeks, each patient and caregiver also received an individual appointment with nursing and medical staff, the pharmacist and the physiotherapist.

Over the fourth and fifth weeks patients and caregivers had group sessions with their peers. Group sessions included: clinical psychology sessions which focused on coping skills and common reactions to recovery from critical illness. On the final (fifth) week; the social prescription week, social problems which individuals may have been experiencing were explored.

InS:PIRE was facilitated by a multi-professional team including a trained ICU Nurse, Physician, Physiotherapist and Pharmacist. A Consultant Clinical Psychologist provided psychological care and input as appropriate.

**Fig 1:**
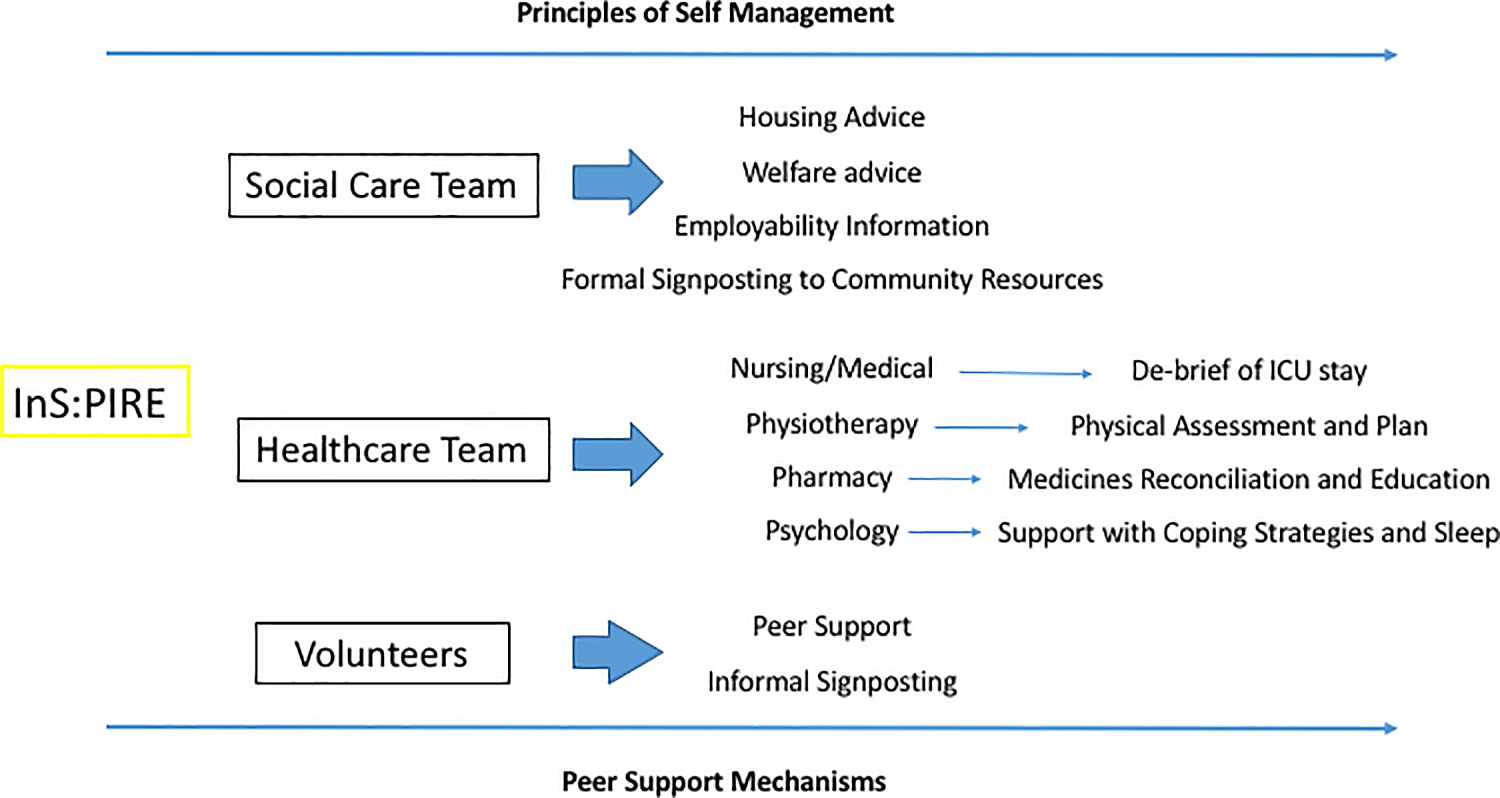
InS:PIRE conceptual framework.

Peer support was developed within each cohort by the patients and caregivers taking part. Peer support also came from patients and caregiver volunteers who were further along the recovery trajectory; they ran a social café area for participants. A conceptual framework of the InS:PIRE intervention is detailed in **Fig 1**.

After each cohort, a learning session took place with the Multi-Disciplinary team (MDT) to understand how improvements could be implemented. Feedback was also obtained from the participants to feed into learning sessions. These changes improved the quality of the programme as opposed to the content. For example, we added in strategies such as texting patients on the morning of the clinic as a reminder, which helped those with cognitive impairment.

### Historical controls

We compared those who participated in InS:PIRE with a historical control group from the same centre who had not participated. The control cohort were 52 working age patients admitted to the GRI ICU between 2008–2009. These patients completed the EQ-5D after hospital discharge as part of an observational cohort study exploring social dependency following ICU. Data on this group has been previously published [12]. This group were chosen as they had similar data available to allow for a holistic comparison. Self-Efficacy measures were not available for the historical control cohort.

### Measures

Quality of life was measured using the EQ-5D 3L tool (EuroQuality of Life Group) [25]. This tool comprises two sections: a five question descriptive component which explores various health domains and a visual analogue scale about the quality of life on the day the questionnaire was completed. Each of the five questions has three possible answers. These answers equate to a five digit sequence which is then used to determine a Health Utility Score (HUS). In EQ-5D evaluations, a HUS of 1 equates to the best health state possible, 0 with death and a negative HUS equates to a state worse than death [25]. The EQ-5D was obtained from patients at both baseline (during the initial five week intervention) and at one year. Based on previous literature, the Minimally Important Clinical Difference(MCID) for the HUS for critical care and the UK time-trade-off “tariff,” is approximately 0.08 [26–27].

To measure empowerment in this patient cohort, the Generalised Self-Efficacy tool, which uses a 10 point questionnaire form, was administered to patients. The scale ranges from 10–40 (10 lowest possible self-efficacy and 40 being the highest achievable score) [28]. Self-efficacy measurements were undertaken at baseline (start of the five week intervention), week five (end of five week intervention) and at 12 months. Minimally clinically important differences (MCID) for self-efficacy are poorly documented in the literature. An adapted Generalised Self Efficacy tool demonstrated a 5.8–6.9% change after a pulmonary rehabilitation intervention for patients with COPD, a service which has been widely adopted and recommended in the UK [29–30]. In this study, we took a 2.4 (6%) score change in self-efficacy to be clinically meaningful. We also examined the relationship between self-efficacy scores and HUS at one year within this analysis.

Finally, this evaluation sought to understand the impact, if any, that the InS:PIRE programme had on return to employment relative to the historical control group [12]. This outcome was collected as a binary measure. Patients were asked at their one year follow up appointment if they were employed or undertaking volunteering roles. Only information on paid employment was available for the historical control cohort; information on volunteering roles was not available or included for the historical control group.

A qualitative analysis, by an independent clinician, was undertaken to understand the potential benefit of the programme. Semi structured interviews were undertaken at six months with 11 patients and caregivers. Patients and caregivers were purposively sampled to undertake these interviews. All interviews were audio recorded and transcribed verbatim. Content analysis using Burnard’s approach was utilised [31]. An audit trail and peer review by external researchers was utilised to ensure the credibility of the findings.

The Scottish Index of Multiple Deprivation (SIMD) is the Scottish Government’s tool for identifying those geographical areas in Scotland suffering from deprivation. With a research context, the SIMD data is split into quintiles, deciles or vintiles. For the purpose of this evaluation, deciles were utilised, with decile one being the most deprived and decile 10 being the most affluent [23].

### Statistical analysis

Data was analysed using the statistical package R (Version 3.3.0) [32]. Continuous variables were expressed as medians and inter quartile ranges (IQR) or means and ranges and analysed using the Mann-Whitney *U* test or the two sample t-test. Categorical variables were compared using chi squared tests. All tests were two sided and a *p* value of less than 0.05 was considered significant.

Multivariable regression to adjust for differences between the groups (historical control vs. InS:PIRE cohort) was also utilised. Three approaches were produced, as there was not an a priori-specified analysis plan, nor is there clear consensus about the best way to select for possible confounders when there is limited sample size. First, we measured the differences between the two groups when those factors known, based on the literature and clinical judgement, to impact long term outcomes from critical care were included [33–34]. A second controlled for all variables which were significantly different in an unadjusted analysis between the two groups, using a criteria of p<0.01 in the bivariate associations for inclusion in the final model. A third utilised Backward Stepwise Regression to identify the best fitting model with the covariates which were available for both datasets. Seven patients had one missing data point and were included using multiple imputation [35].

After external review, we also undertook the genetic propensity matching approach, to try and adjust for the imbalances between the two groups [36].

## Results

### Demographics

89 patients were invited to participate in InS:PIRE. 49 (55% of those invited) patients attended over the one year evaluation. The 40 patients who received the InS:PIRE intervention did not attend for any form of intervention or follow up. Two patients were older than working age and were excluded from analysis. Only one patient who attended the programme did not receive all of the core interventions (98% of patients who started the programme received all core interventions); this patient has also been excluded from analysis.

**Fig 2:**
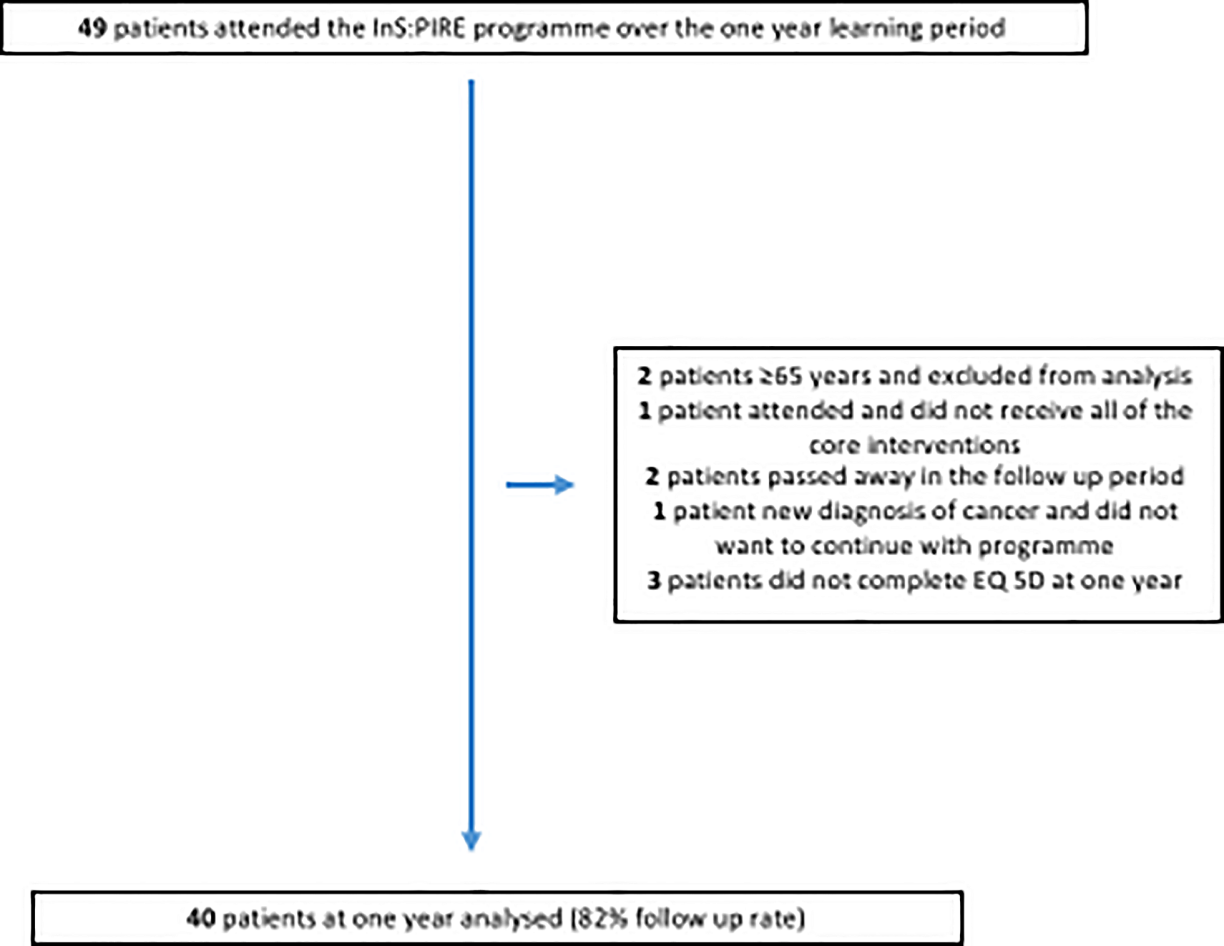
Patient flow through the InS:PIRE programme.

Over the one year follow up period, two patients died and one patient was diagnosed with cancer and asked not to continue with follow up. Three other patients were lost to follow up. 40 patients are included in the analysis of the InS:PIRE cohort **(Fig 2)**, a follow up rate of 82% at one year.

Baseline demographics for the cohort of patients who completed the InS:PIRE programme are shown in **Table 1**. In this cohort, 62.5% were male and the median length of ICU stay was 15 days (IQR 9–27 days).

**Table 1:** Baseline demographics from the InS:PIRE and historical control groups.

Patient Characteristic	InS:PIRE cohort (n = 40)	Historical Control (n = 52)	p value
*Gender (male %)*	62.5	59.6	0.95
*Age (years*, *median*, *IQR)*	51 (43–57)	46.5 (40–52)	0.13
*ICU LOS (days*, *median*, *IQR)*	15 (9–27)	3 (1–10)	<0.001
*APACHE II (median*, *IQR)*	23 (19–27)	14 (9–19)	<0.001
*Charlson Co-Morbidity Index (median*, *IQR)*	1 (0–2)	0 (0–2)	0.32
*Patients with Mental Health Problems Pre ICU (%)*	42.5	40.4	1
*SIMD Decile (median*, *IQR)*	3 (1–4)	2 (1–5.5)	0.27
*Hospital LOS (days*, *median*, *IQR)*	49 (22–80)	24 (11–51)	0.004
*Proportion Ventilated (%)*	95	78.8	0.08
*Proportion undergoing RRT (%)*	35	13.5	0.03
*Proportion undergoing CVS (%)*	50	17.3	0.002
*Unemployed Pre-ICU admission (%)*	42.5	26.9	0.15
*Unemployed Post-ICU*	60	48.1	0.36
*HUS (Median*, *IQR) at one year*	0.62 (0.32–0.73)	0.52 (0.02–0.71)	0.27
*Follow up time for EQ-5D completion (days*, *median*, *IQR)*	487 (462–608)	813 (737–880)	<0.001

IQR: Interquartile Range; ICU LOS: Intensive Care Unit Length of Stay; APACHE II: Acute Physiology and Chronic Health Evaluation II; SIMD: Scottish Index of Multiple Deprivation; Hospital LOS: Hospital Length of Stay; RRT: Renal Replacement Therapy; CVS: Cardiovascular Support; HUS: Health Utility Score (EQ-5D Quality of Life measure)

Baseline demographics for the historical control group are also displayed in **Table 1**. In this cohort 59.6% were male, the median length of ICU stay was 3 days (IQR 1–10 days).

### Quality of life

There was a significant change in HUS as measured with the EQ-5D between baseline and one year in the InS:PIRE cohort. The median HUS increased from 0.29 (IQR -0.03–0.62) to 0.62 (IQR 0.32–0.73) (p = 0.009). Some improvement in EQ-5D is expected over time, therefore, the InS:PIRE cohort was compared to a historical control group. The demographics of this historical control group, alongside the intervention group from InS:PIRE are given in **Table 1**.

There were several differences between the InS:PIRE cohort and the historical controls. The InS:PIRE cohort had substantially longer ICU lengths of stay (median: 15 days vs. 3 days); higher APACHE II scores (median 23 vs. 14), and higher levels of ICU support including ventilation and RRT. They were also followed up sooner after ICU discharge (1.3 years after discharge versus 2.2 years). Despite these differences, the HUS was not statistically different between the InS:PIRE cohort and the historical controls (InS:PIRE 0.62 vs. controls 0.52, p = 0.27).

**Fig 3:**
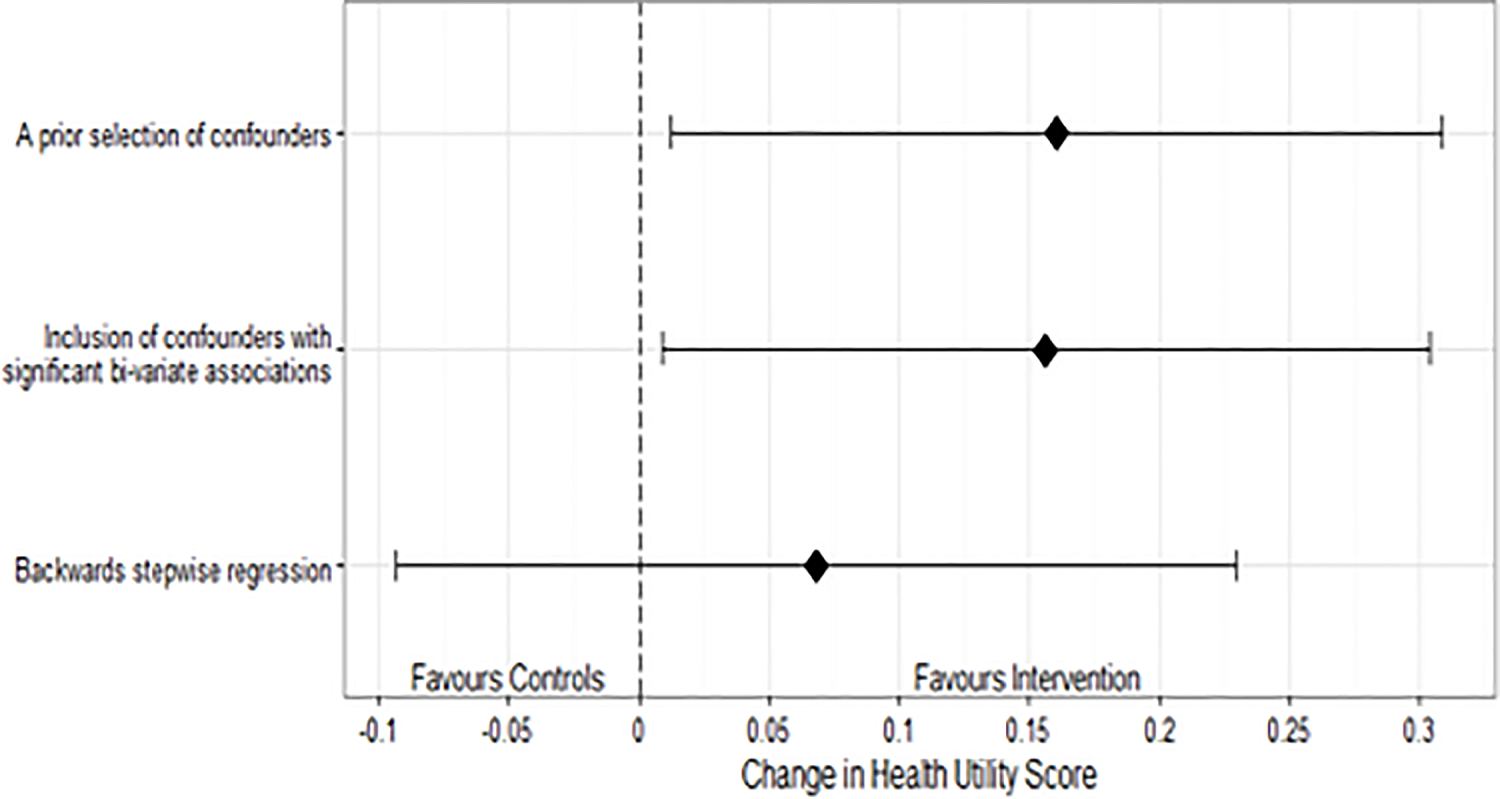
Forest plot demonstrating the estimate and confidence intervals of each model.

In order to adjust for these imbalances, we used three different regression adjustment strategies. Multiple approaches were necessary as the number of potential confounders was large relative to the cohort sizes. Full results for each model are presented in S1 File. In an analysis controlling for a priori of selected confounders, based on the past literature, InS:PIRE was associated with a 0.16 increase in HUS (95% CI 0.01–0.31; p = 0.03) versus what would have been expected based on the historical controls. In an analysis where covariates were selected based on their bi-variate association with the outcome, InS:PIRE was associated with a with a 0.16 increase in HUS (95% CI 0.01–0.30; p = 0.04). In an analysis where covariates were selected by backwards stepwise regression, InS:PIRE was associated with a 0.07 increase in HUS (95% CI -0.09–0.23; p = 0.40) relative to historical controls **(Fig 3)**.

Using a genetic propensity matching approach on both gender and age, 37 out of the 50 controls were accurately matched to the 40 in the treatment group. The overall percentage balance improvement in matching was 97.4%, with the addition of age improving by 95.8% and gender by 100%. Both the clinically specified unadjusted-adjusted models on the propensity matched data show that there is a statistically significant difference in health utility scores at one year between the InS:PIRE group and historical control group (S1 File).

### Self-efficacy

**Fig 4:**
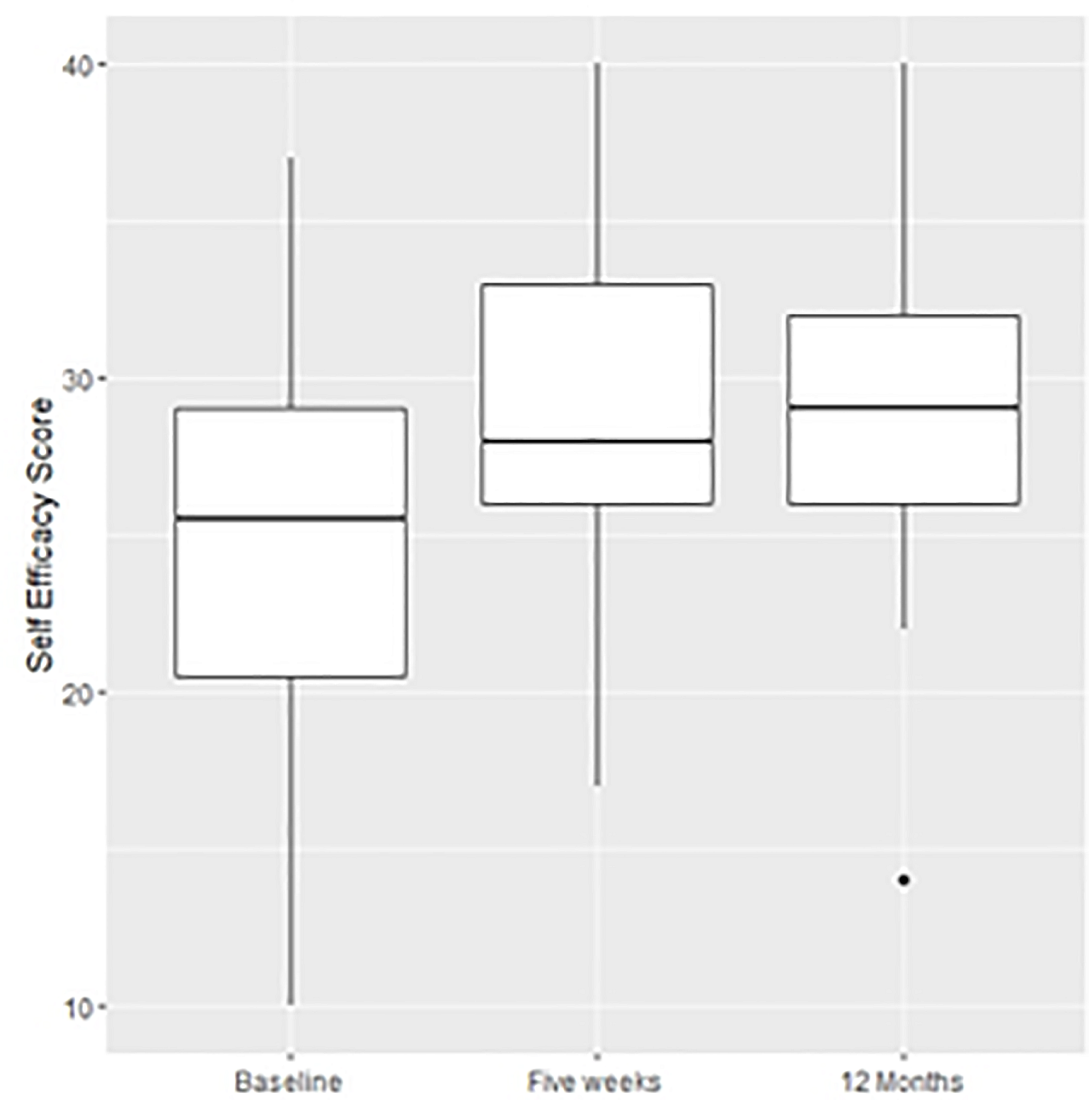
Boxplot demonstrating the change in self-efficacy scores over the one year evaluation period.

Self-efficacy scores increased significantly from a median of 25.5 (IQR 20.5–29) at baseline to 28 (IQR 26–33) at five weeks (p = 0.003). This represented a change of 2.5 points (6.25% change) over the five week period. This change was sustained at one year post InS:PIRE, with patients having a median self-efficacy score of 29 (IQR 26–32) **(Fig 4)**.

At one year, there was a relationship between self-efficacy scores and the HUS. With every one point increase in self-efficacy, the HUS increased by 0.025 (95% CI 0.007–0.04; p = 0.008).

### Return to employment

In the InS:PIRE cohort 17 of the 40 (42.5%) patients were employed prior to ICU. Four were retired (12.5%) and 19 were unemployed or chronically unwell pre ICU (42.5%). In the year following ICU, 15 InS:PIRE patients returned to employment or volunteering roles (88%) compared with 11 (46%) in the historical control group (p = 0.15).

### Qualitative evaluation

11 patients and caregivers took part in interviews with an independent clinician at six months post intervention to understand the individual benefits of taking part. The themes and sub themes generated are shown in **Table 2**.

**Table 2:** Themes and sub themes generated from the qualitative interviews.

Themes	Sub Themes	Supporting quotes
Support	• Patient Volunteers provided hope, optimism and peer support • Cohesive Approach by staff • Created a community	*‘It’s good to see how far somebody who has been so ill*, *like yourself*, *can improve and recover over a period of time*.*’*
Psychological Impact	• Understanding of symptoms and coping mechanisms • Family benefit • Feeling normal, acknowledgment of illness	*‘*.*it was so good to know that you were normal*. *I didn’t feel like an abnormal person anymore*, *I felt like everything I was feeling was a normal reaction*.*’*
Physical Impact	• Increased confidence and independence • Use of goals important • Importance of involving carers	*‘I was nervous in case I didn’t achieve them and then coming back today to realise that I have actually overachieved a couple…*.*it was a good thing because I would not have set goals for myself’*.
Future Direction	• Longer time/ balanced with dependency • Importance of follow up	*‘Slightly longer- I would have thought about 8 weeks*.*’*

## Discussion

In this initial evaluation of a complex intervention for ICU survivors, 98% of those who started the program received all of the core interventions. The present data suggest that, after adjustment, InS:PIRE improves quality of life compared with a historical control group. InS:PIRE patients also experienced a clinically meaningful improvement in self-efficacy scores. Qualitative interviews in selected patients and caregivers painted a complementary picture of benefit.

Studies have previously demonstrated the social impact of critical illness for both patients and their loved ones [12–13]. InS:PIRE is one of the first rehabilitation programmes for this population which has included both health and social care support. This may be synergistic for optimal recovery for this patient group [37]. Further, the use of peer support as a mechanism for informal support also appears to have made a significant impact on the recovery trajectory of this population.

Three analysis strategies were utilised to measure the impact of InS:PIRE on Health Utility. All three analyses demonstrated a consistent direction of positive change in outcomes for those who did receive InS:PIRE compared to those who did not. These adjusted analyses were necessary because the best available historical controls—although from the same institution—were not randomly assigned to insure potential confounder balance. Historical controls have a wide-range of known weaknesses and so this association should be interpreted cautiously [38]. We believe it provides evidence for more careful testing and certainly does not constitute proof of efficacy at this stage.

This is one of the first studies which has explored the concept of self-efficacy in survivors of critical illness. Self-efficacy in this context is defined as a person’s confidence or belief in their ability to undertake a certain set of actions [39]. This study is consistent with previous work which has linked self-efficacy with improved recovery and quality of life from disease processes [40]. When compared to another study investigating the effect of pulmonary rehabilitation on self-efficacy, InS:PIRE appears to have an impact of the same magnitude [29]. We hypothesize that improved self-efficacy is an important mechanism which can lead to improved long term outcomes. Improved self-efficacy might do this by increasing confidence and self-esteem.

This study demonstrated that a higher proportion of patients returned to employment in the InS:PIRE cohort compared the to the historical control group. There was parity of focus on health and social care within the InS:PIRE programme which helped support this element of rehabilitation. Furthermore, the link between employment and quality of life is well known, thus may have impacted upon the improved HUS seen in the InS:PIRE cohort [41]. If replicated in a larger analysis, the improvement seen in this initial evaluation, has the potential to have a significant impact on social care costs.

## Limitations

This early evaluation has a number of limitations. Firstly, the InS:PIRE intervention was undertaken as part of a learning project and utilised a historical control group to understand the potential impact of this intervention. This historical control group is much older and is distinctly different from the InS:PIRE cohort. Utilising a RCT to undertake the evaluation for InS:PIRE will be necessary for a pivotal demonstration of efficacy; the work presented here is better thought of as earlier testing, e.g. Phase I-II. Further, this evaluation took place in a single centre in an area of high deprivation. Future work should analyse the impact of this intervention across several sites. A further limitation of the study is that details of the patient’s pre-hospital quality of life were not available. As a result, despite the increase in EQ-5D which was demonstrated in this study, we are unsure if this is In- line with the patients pre-ICU quality of life. Future studies should aim, if possible to understand this. Finally, there was some loss to follow-up, which could introduce bias if it was differential.

## Conclusion

This evaluation has demonstrated that the delivery of a multi-faceted intervention for ICU survivors is feasible. It has also suggested that this multi-faceted intervention may improve functional quality of life for this patient group. A further, large multi-centre study is required to evaluate the full impact of this intervention.

## Declarations

We have all necessary consent for publication

Disclaimer:

This does not necessarily represent the official views of the US Government or Department of Veterans Affairs.

The Health Foundation is a charitable organisation. They had no role in the design, collection, analysis, interpretation of data or in the writing of this manuscript. They had no role in the decision to submit the manuscript for publication.

## Supporting information

S1 File Modelling strategies utilised. (DOCX)

## Abbreviations

APACHE II: Acute Physiology and Chronic Health Evaluation II
GRI: Glasgow Royal Infirmary
HUS: Health Utility Score
ICU: Intensive Care Unit
InS:PIRE: Intensive Care Syndrome: Promoting Independence and Return to Employment
IQR: Inter quartile Range
MCID: Minimally Clinically Important Difference
SIMD: Scottish Index of Multiple Deprivation
