# Supplementary material for: Intensive Care Syndrome: Promoting Independence and Return to Employment (InS:PIRE). Early evaluation of a complex intervention
Source: PLoS One. 2017 Nov 29;12(11):e0188028. doi: 10.1371/journal.pone.0188028 (PMC5706708; doi:10.1371/journal.pone.0188028)
Supplement: S1 File — (DOCX) [file pone.0188028.s001.docx]

**Supplementary File One**

*Model One*

The first modelling strategy corrected for covariates which are known to impact long term outcomes from critical care (SIMD, Charlson Co-morbidity, in work pre-ICU) (p=0.0006, r²=0.17).

For those who undertook the InS:PIRE programme there was a significant increase in their HUS after adjustments for these covariates (0.16; 95% CI 0.01-0.31; p=0.03) **(Table One)**.

| **Variable Estimate** | | **p value** |
| --- | --- | --- |
| *Charlson Co-Morbidity Index* | -0.06 (-0.11- -0.01) | 0.02 |
| *SIMD decile* | 0.03 (0.01-0.06) | 0.02 |
| *Unemployed Pre ICU* | -0.18 (-0.33- -0.02) | 0.03 |

**Table One: Association between Charlson Score, SIMD decile and Employment pre ICU and HUS at one year**

*Model Two*

In Model Two, we undertook univariate modelling for all covariates which were available for both datasets. The significant covariates were then entered into a multivariable linear model (p=0.0009, r²=0.18) **(Table Two)**. The cohort covariate (the variable utilised to separate the historical control and InS:PIRE cohort) was not significant in the unadjusted analysis. However, it was included in the multivariable analysis as this was how we tested for cohort difference.

|  | **Unadjusted Value Adjusted Value** | | | |
| --- | --- | --- | --- | --- |
| **Variable** | **Estimate p value** | | **Estimate p value** | |
| *Cohort* | 0.09 (-0.06-0.25) | 0.23 | 0.16 (0.009-0.30) | **0.04** |
| *Gender (male)* | -0.09(-0.24-0.07) | 0.28 |  |  |
| *Age (years)* | -0.003 (-0011-0.005) | 0.41 |  |  |
| *ICU LOS (Days)* | 0.0003(-0.005-0.005) | 0.89 |  |  |
| *APACHE II* | 0.004 (-0.006-0.013) | 0.423 |  |  |
| *Charlson Co-morbidity Index* | -0.05 ( | **0.05** | -0.05(-0.11- -0.006) | 0.06 |
| *Mental Health Problems Pre-ICU* | -0.22 (-0.37- -0.07) | **0.005** | -0.1(-0.28-0.05) | 0.24 |
| *SIMD decile* | 0.04 (0.01-0.06) | **0.008** | 0.03 (-0.001-0.05) | 0.06 |
| *Hospital LOS (Days)* | -0.004 (-0.002-0.001) | 0.67 |  |  |
| *Ventilated* | -0.05 (-0.28-0.18) | 0.65 |  |  |
| *RRT* | 0.05(-0.14-0.24) | 0.59 |  |  |
| *CVS* | -0.02 (-0.18-0.15) | 0.85 |  |  |
| *Unemployed Pre-ICU* | -0.19 (-0.35- -0.30) | **0.02** | -0.08(-0.031-0.03) | 0.44 |

**Table Two: Adjusted and unadjusted difference between cohorts.**

*Model Three*

Similar to Model Two, the cohort covariate was reintroduced into the Backward Stepwise Regression. Utilising this approach, a model including APACHE II, Charlson Co-morbidity Index, SIMD Decile and Unemployment pre ICU was produced (p<0.001, r²=0.17) **(Table Three)**.

| **Variable** | **Estimate** | **p value** |
| --- | --- | --- |
| *Cohort* | 0.07 (-0.001-0.23) | **0.40** |
| *APACHE II* | 0.01(-0.001-0.02) | 0.05 |
| *Charlson Co morbidity Index* | -0.06(-0.001- -0.008) | 0.02 |
| *SIMD Decile* | 0.03 (9.20-0.06) | 0.008 |
| *Unemployed Pre-ICU* | -0.18(--3.27- -0.02) | 0.002 |

**Table Three: Backward Stepwise Regression Model**

**Propensity matched Models**

| **Patient Characteristic** | **InS:PIRE cohort (n=40)** | **Historical Control**  **(n=34)** | **p value** | **Weighted**  **Mean**  **Standardised**  **Difference** |
| --- | --- | --- | --- | --- |
| *Gender (male %)* | 62.5 | 67.6 | 0.828 | 0% |
| *Age (years, median, IQR)* | 51 (43-57) | 46.5 (43.25-56) | 0.441 | -0.017 |
| *ICU LOS (days, median, IQR)* | 15 (9-27) | 2.5 (1-8.25) | <0.001 | 0.886 |
| *APACHE II (median, IQR)* | 23 (19-27) | 14 (8.5-18.5) | <0.001 | 1.024 |
| *Charlson Co-Morbidity Index (median, IQR)* | 1 (0-2) | 1 (0-2) | 0.449 | 0.056 |
| *Patients with Mental Health Problems Pre ICU (%)* | 42.5 | 35.3 | 0.694 | 0% |
| *SIMD Decile (median, IQR)* | 3 (1-4) | 2 (1-6.75) | 0.102 | -0.324 |
| *Hospital LOS (days, median, IQR)* | 49 (22-80) | 24 (12-50) | 0.009 | 0.527 |
| *Proportion Ventilated (%)* | 95 | 73.5 | 0.042 | 19.5% |
| *Proportion undergoing RRT (%)* | 35 | 11.8 | 0.047 | 25.7% |
| *Proportion undergoing CVS (%)* | 50 | 17.3 | 0.002 | 32.7% |
| *Unemployed Pre-ICU admission (%)* | 42.5 | 26.5 | 0.201 | 25.7% |
| *Unemployed Post-ICU* | 60 | 44.1 | 0.258 | 22.9% |
| *HUS (Median, IQR) at one year* | 0.62 (0.32-0.73) | 0.516 (-0.003-0.71) | 0.291 | 0.31 |
| *Follow up time for EQ-5D completion (days, median, IQR)* | 487 (462-608) | 825.5 (772.5-895.75) | <0.001 | -1.97 |

Table Four: Individual contribution for each subject weighted according to the genetic propensity matching.

*Propensity Matched Model One*

The first modelling strategy corrected for covariates which are known to impact long term outcomes from critical care (SIMD, Charlson Co-morbidity, in work pre-ICU) (p<0.001, r²=0.185).

For those who undertook the InS:PIRE programme there was a significant increase in their HUS after adjustments for these covariates (0.16; 95% CI 0.003-0.32; p=0.05) **(Table Five)**.

| **Variable Estimate** | | **p value** |
| --- | --- | --- |
| *Charlson Co-Morbidity Index* | -0.06 (-0.11- -0.01) | 0.047 |
| *SIMD decile* | 0.03 (0.004-0.06) | 0.027 |
| *Unemployed Pre ICU* | -0.19 (-0.36- -0.02) | 0.034 |

**Table Five: Association between Charlson Score, SIMD decile and Employment pre ICU and HUS at one year**

*Propensity matched Model Two*

In Model Two, we undertook univariate modelling for all covariates which were available for both datasets. The significant covariates were then entered into a multivariable linear model (p<0.001, r²=0.18) **(Table Two)**. The cohort covariate (the variable utilised to separate the historical control and InS:PIRE cohort) was not significant in the unadjusted analysis. However, it was included in the multivariable analysis as this was how we tested for cohort difference.

|  | **Unadjusted Value Adjusted Value** | | | |
| --- | --- | --- | --- | --- |
| **Variable** | **Estimate p value** | | **Estimate p value** | |
| *Cohort* | 0.104 (-0.070 - 0.278) | 0.238 | 0.16 (0.003-0.32) | **0.05** |
| *Gender (male)* | -0.038 (-0.222 - 0.146) | 0.678 |  |  |
| *Age (years)* | -0.001 (-0.011 - 0.008) | 0.769 |  |  |
| *ICU LOS (Days)* | 0.0004 (-0.005 - 0.006) | 0.895 |  |  |
| *APACHE II* | 0.003 (-0.008 - 0.014) | 0.601 |  |  |
| *Charlson Co-morbidity Index* | -0.056 (-0.115 - 0.003) | **0.064** | -0.05(-0.11- -0.002) | 0.07 |
| *Mental Health Problems Pre-ICU* | -0.222 (-0.394 - -0.050) | **0.012** | -0.07(-0.26-0.11) | 0.44 |
| *SIMD decile* | 0.038 (0.009 0.067) | **0.011** | 0.03 (-0.001-0.06) | 0.07 |
| *Hospital LOS (Days)* | -0.001 (-0.003 - 0.001) | 0.471 |  |  |
| *Ventilated* | -0.046 (-0.299 - 0.208) | 0.721 |  |  |
| *RRT* | 0.033 (-0.169 - 0.235) | 0.746 |  |  |
| *CVS* | -0.016 (-0.200 - 0.169) | 0.867 |  |  |
| *Unemployed Pre-ICU* | -0.231 (-0.407 - -0.055) | **0.011** | -0.17(-0.35-0.02) | 0.08 |

**Table Six: Adjusted and unadjusted difference between cohorts.**

*Propensity matched Model Three*

Similar to Model Two, the cohort covariate was reintroduced into the Backward Stepwise Regression. Utilising this approach, a model including APACHE II, Charlson Co-morbidity Index, SIMD Decile and Unemployment pre ICU was produced (p<0.001, r²=0.18) **(Table Three)**.

| **Variable** | **Estimate** | **p value** |
| --- | --- | --- |
| *Cohort* | 0.096 (-0.089-0.281) | **0.31** |
| *APACHE II* | 0.007(-0.004-0.019) | 0.23 |
| *Charlson Co morbidity Index* | -0.067(-0.124- -0.01) | 0.02 |
| *SIMD Decile* | 0.036 (0.007-0.066) | 0.02 |
| *Unemployed Pre-ICU* | -0.179(--3.53- -0.005) | 0.048 |

**Table Seven: Backward Stepwise Regression Model**
